# Reduced Functional Measure of Cardiovascular Reserve Predicts Admission to Critical Care Unit following Kidney Transplantation

**DOI:** 10.1371/journal.pone.0064335

**Published:** 2013-05-27

**Authors:** Stephen M. S. Ting, Hasan Iqbal, Thomas Hamborg, Chris H. E. Imray, Susan Hewins, Prithwish Banerjee, Rosemary Bland, Robert Higgins, Daniel Zehnder

**Affiliations:** 1 Department of Renal Medicine and Transplantation, University Hospitals Coventry and Warwickshire NHS Trust, Coventry, United Kingdom; 2 Department of Cardiology, University Hospitals Coventry and Warwickshire NHS Trust, Coventry, United Kingdom; 3 Department of Vascular and Renal Transplantation Surgery, University Hospitals Coventry and Warwickshire NHS Trust, Coventry, United Kingdom; 4 Division of Health Sciences Statistics and Epidemiology, Warwick Medical School, The University of Warwick, Coventry, United Kingdom; 5 Division of Metabolic and Vascular Health, Warwick Medical School, The University of Warwick, Coventry, United Kingdom; University of Colorado, United States of America

## Abstract

**Background:**

There is currently no effective preoperative assessment for patients undergoing kidney transplantation that is able to identify those at high perioperative risk requiring admission to critical care unit (CCU). We sought to determine if functional measures of cardiovascular reserve, in particular the anaerobic threshold (VO_2_AT) could identify these patients.

**Methods:**

Adult patients were assessed within 4 weeks prior to kidney transplantation in a University hospital with a 37-bed CCU, between April 2010 and June 2012. Cardiopulmonary exercise testing (CPET), echocardiography and arterial applanation tonometry were performed.

**Results:**

There were 70 participants (age 41.7±14.5 years, 60% male, 91.4% living donor kidney recipients, 23.4% were desensitized). 14 patients (20%) required escalation of care from the ward to CCU following transplantation. Reduced anaerobic threshold (VO_2_AT) was the most significant predictor, independently (OR = 0.43; 95% CI 0.27–0.68; p<0.001) and in the multivariate logistic regression analysis (adjusted OR = 0.26; 95% CI 0.12–0.59; p = 0.001). The area under the receiver-operating-characteristic curve was 0.93, based on a risk prediction model that incorporated VO_2_AT, body mass index and desensitization status. Neither echocardiographic nor measures of aortic compliance were significantly associated with CCU admission.

**Conclusions:**

To our knowledge, this is the first prospective observational study to demonstrate the usefulness of CPET as a preoperative risk stratification tool for patients undergoing kidney transplantation. The study suggests that VO_2_AT has the potential to predict perioperative morbidity in kidney transplant recipients.

## Introduction

A successful kidney transplant reduces the cardiovascular mortality risk when compared with maintenance dialysis therapy and relies on survival beyond the perioperative period [1], [2]. Currently, there is no definitive consensus on the optimal pre-kidney transplant cardiac assessment strategy to produce a reliable prediction of perioperative morbidity following kidney transplantation. Intraoperative oxidative stress during surgery induces an increase in myocardial oxygen demand that is further exacerbated by perioperative fluid shift [3]. Patients with kidney failure have pre-existing co-morbid illness that contributes to structural cardiac and vascular alterations (myocardial fibrosis, excessive arterial calcification, intimal hyperplasia) [4], [5]. These complex cardiovascular alterations affect microvascular flow and myocardial turgor, thereby are not conducive in augmenting stroke volume or sustaining large volume cardiac output [6].

The main novelty of cardiopulmonary exercise testing (CPET) lies in its ability objectively to measure cardiovascular functional reserve, in contrast to other tools frequently used to assess cardiac function such as echocardiography or cardiac magnetic resonance (CMR) which focus mainly on left ventricular (LV) morphology [7]–[9]. CPET induces cardiovascular stress through incremental level of work and determines the ability of the cardiovascular system to drive and sustain the coupling between the cellular and pulmonary respirations under stress conditions [10]–[12]. The potential diagnostic utility of CPET for exercise-induced myocardial ischemia causing LV dysfunction has also been demonstrated in previous studies [13], [14]. Therefore, the large amount of data generated by CPET could be used objectively to evaluate cardiovascular response to physiologically induced stress.

Several studies have demonstrated that non-invasive CPET derived measures of cardiovascular reserve, especially the anaerobic threshold (VO_2_AT) provide a reliable predictor of perioperative complications and mortality following major intra-abdominal surgery [15]–[18]. However, there is currently no such effective preoperative risk stratifying assessment for kidney transplant recipients. The goal of this prospective study is to examine the usefulness of CPET as a risk stratification tool for patients undergoing kidney transplantation. We hypothesized that functional quantification of cardiovascular reserve will identify kidney transplant recipients who have a high risk of developing perioperative complication requiring critical care unit (CCU) support following surgery.

## Materials and Methods

### Ethics Statement

The study protocol was approved by the Black Country Research Ethics Committee (REC reference number: 09/H1202/113).

### Study Design

All patients aged 18 or over undergoing kidney transplantation at the University Hospitals Coventry and Warwickshire NHS Trust (United Kingdom) were invited to participate within 4 weeks prior to the surgery between April 2010 and June 2012. These patients had previously undergone standard kidney transplant work-up according to our center’s protocol that included clinical evaluation, chest X-ray, electrocardiogram and echocardiography. Each prospective transplant recipient was evaluated by a transplant physician and a transplant surgeon. Thallium myocardial perfusion imaging, exercise tolerance test, coronary angiography, pulmonary function test and imaging of the iliac vessels were performed selectively as determined by the clinical assessment during the work-up period. All transplant recipients were reviewed by the anesthetist 6–36 hours prior to surgery.

For the purpose of this study, all recruited patients underwent the additional CPET, arterial applanation tonometry and a study-specified echocardiography within 4 weeks prior to transplantation. CPET data were not used to determine the inclusion or exclusion of prospective kidney transplant recipients. All assessments at study entry were carried out on the first non-dialysis day for living donor kidney transplant recipients who were hemodialysis dependent and prior to first antibody removal therapy with double filtration plasmapheresis (desensitization) for those who had donor kidney directed antibodies [19]. Recipients of kidneys from deceased donors were approached on the day of admission and assessed prior to surgery. All participants were recruited as part of an ongoing prospective, longitudinal, non-randomized concurrent control study, designed to determine the impact of kidney transplantation on functional cardiovascular reserve and LV dimensions, whereby patients were re-evaluated with CPET and echocardiography at 60 days and 1 year following transplantation. Patients with conditions precluding exercise testing were excluded. Written informed consent was obtained from all eligible participants.

The main outcome of interest was unplanned admission to CCU due to post-operative requirement for invasive or intensive central hemodynamic monitoring allowing for accurate infusion of volume expanders or circulatory support with vasoactive drugs. Indications for this were persisting hemodynamic instability alone or hemodynamic instability in patient with sepsis. Hemodynamic instability was defined as persistently low systolic arterial blood pressure (≤85 mmHg) despite adequate blood volume expansion targeted to a central venous pressure of 10–12 mmHg and as determined by clinicians in the ward. In addition, an observation based modified early warning scoring system (MEWS) [20], [21] was used to trigger early assessment and intervention on the ward. Involved clinicians were blinded to the CPET results.

### Clinical Data

Baseline clinical and demographic data were recorded including weight, height, body mass index (BMI), duration of CKD and dialysis vintage. Assessment of traditional cardiovascular risk factors included a history of any of the following: *(1)* A previous coronary artery disease event (CAD: nonfatal myocardial infarction, acute coronary syndrome requiring hospitalization, percutaneous coronary intervention or coronary artery bypass graft), *(2)* cerebrovascular disease (CVD: transient ischemic attack or stroke), *(3)* diabetes (use of oral hypoglycemics or insulin), *(4)* dyslipidemia (use of lipid-lowering therapy), *(5)* hypertension (use of antihypertensives), and *(6)* smoking status (ever or never). Serial 12-lead electrocardiograms were performed at study entry, during hospital admission for surgery (including the pre-, peri- and postoperative period as well as during stay in the CCU) and at clinic visit, 60 days following transplantation. All kidney transplant recipients who were admitted to CCU were identified. Length of stay in the CCU and hospital were recorded.

### Cardiopulmonary Exercise Testing

Prior to exercise testing, forced expiratory volume in 1 sec (FEV_1_) and full vital capacity (FVC) of the lungs were measured. Exercise testing to maximal exhaustion using cycle ergometer ramping protocol, incorporating an individualized work rate was performed. Minute ventilation (VE), oxygen consumption (VO_2_), carbon dioxide production (VCO_2_) and other associated parameters were acquired via breath-by-breath analysis (VIASYS, MasterScreen CPX®, Hoechberg, Germany). The metabolic cart was recalibrated for consecutive patients. A 12-lead electrocardiogram was recorded continuously throughout testing and during recovery. Test was terminated at maximal exhaustion, accompanied by attainment of a respiratory exchange ratio (RER, ratio of CO_2_ production to O_2_ consumption) of a value ≥1.15. VO_2_peak was measured as the highest VO_2_ achieved during the final 30 sec of peak exercise. VO_2_AT was determined by the V-slope method in conjunction with analyses of the ventilatory equivalents (VE/VO_2_ and VE/VCO_2_) and end-tidal gas tensions (P_ET_O_2_ and P_ET_CO_2_) plots [22]. This was undertaken by two experienced investigators.

### Echocardiographic Study

2-dimensional echocardiography and Doppler blood flow measurements were performed [23]. Calculations included left LV diastolic and systolic volumes, LV mass, ejection fraction (LVEF) according to Simpson’s method and left atrial volume (LAV). Mitral inflow measurements included peak early (E) and late (A) flow velocities. Tissue Doppler imaging of the mitral annulus, sequentially at the lateral and septal annular sites were obtained from the apical 4-chamber view. The ratio of early transmitral flow velocity to averaged annular (septal and lateral) mitral velocity (E/E’) was taken as an estimate of LV filling pressure.

### Arterial Applanation Tonometry

The SphygmoCor System (AtCor Medical Pty Ltd., Australia) was used to evaluate arterial compliance. Aortic pulse wave-form, augmentation pressure, and augmentation index were derived by tonometric applanation of the radial artery. Using similar technique, carotid-femoral pulse wave velocity (PWV) was determined from the time taken for the arterial pulse to propagate from the carotid to the femoral artery. A minimum of 3 readings were obtained for all parameters.

### Statistical Methods

Results are expressed as mean, median or frequencies with corresponding confidence interval (CI) estimates depending on the distribution and type of the variable. Logistic regression was utilized to calculate odds ratios (ORs) with 95% CI limits for risk factors of CCU admission. Multiple (binary) logistic regression models were fitted to identify a prediction model for CCU admission. For this, risk factors which were significant in the univariate modeling (plus known confounders) were considered for the model allowing multiple predictor variables. Three different effect selection methods (forward selection, backwards elimination and stepwise selection) were used for the multiple logistic regression modeling to evaluate the consistency of results. A p-value <0.05 was required for effect inclusion in the multivariate modeling and an effect elimination criterion of p<0.10 was chosen. To evaluate the accuracy of different VO_2_AT ‘cut-off’ values to predict CCU admission, ordinary and cross validated receiver operating characteristic (ROC) curves and area under curve (AUC) estimates were calculated. The optimal cut-off is estimated based on the Youden index criterion [24] which is optimal in the sense that it provides a score which reflects the intention of maximizing the overall correct classification rate. All analyses were performed using SAS software, version 9.3 (SAS Institute Inc.).

## Results

### Clinical Characteristics

Eighty-one patients were screened for inclusion into the study and seventy individuals were included after the exclusions of eleven (one patient aged <18 years, one had myopathy secondary to mitochondrial cytopathy, one deceased kidney transplant recipient due to inadequate time for complete assessment and eight patients did not provide consent). Analysis of unplanned CCU admitted patients included the fourteen patients who fulfilled the study-defined indications for CCU admission. One patient who was admitted to CCU electively from theatre for a non-study indication (telemetry monitoring due to prior history of ischemic cardiomyopathy) was grouped as ‘non-CCU’.

Baseline characteristics of seventy CKD patients are shown in *Table 1*. 91.4% of patients received kidneys from living donors and 8.6% were deceased kidney transplant recipients. Mean age of all patients was 41.7±14.5 years and 60% were male. Fifteen recipients (23.4%) of antibody-incompatible living-donor kidneys were treated with plasmapheresis based antibody removal protocol. Following transplantation, fourteen patients (20%) required escalation of care from the ward to CCU. Patients admitted to CCU were predominantly female (71.4%) compared to non CCU patients (32.2%) (Difference Δ = 39.2%, 95% CI 16.9–67.9). CCU patients were older (49.9±12.4 years) compared to patients that did not require critical care support (39.8±14.2 years) (Δ = 10.1, 95% CI 2.1–18.7). Forty-five (64.3%) patients were dialysis dependent prior to transplantation and 28.9% of these patients required critical care support following surgery. More than half of those who had antibody removal therapy (57.1%) required CCU support following transplantation (Δ = 44.6%, 95% CI 16.9–67.9). There were no associations between preexisting coronary artery disease and diabetes with CCU admissions.

**Table 1:** Baseline characteristics of the patients.

Characteristics	Non-CCU patients (N = 56)	CCU patients (N = 14)	Difference (95% CI)
Age, years	39.8±14.2	49.9±12.4	10.1 (2.1–18.7)
Male, n (%)	38 (67.8)	4 (28.6)	−39.2 (−59.3– −10.1)^(1)^
Caucasian, n (%)	47 (83.9)	13 (92.8)	8.9 (−22.0–16.5)^(1)^
BMI, kg/m^2^	24.8±3.7	24.6±3.3	−0.2 (−2.4–1.9)
Desensitized, n (%)	7 (12.5)	8 (57.1)	44.6 (17.7–67.0)^(1)^
CKD duration, months	114.0 (46.5–210.3)	200.5 (93.0–270.0)	60.0 (0.0–132.0)^(2)^
Dialysis duration, months	4.5 (0.0–26.8)	32.0 (18.8–89.5)	24.0 (6.0–36.0)^(2)^
Pre-dialysis, n (%)	24 (42.9)	1 (7.2)	−35.7 (−8.6– −50.0)^(1)^
Dialysis-dependent, n (%)	32 (57.1)	13 (92.8)	35.7 (8.6–50.0)^(1)^
Resting blood pressure			
Systolic, mmHg	137.3±20.6	137.6±16.8	0.3 (−11.5–12.3)
Diastolic, mmHg	82.2±12.3	80.4±6.4	−1.8 (−8.7–4.9)
Cardiovascular risk factors			
Hypertension (%)	52 (92.9)	12 (85.7)	−7.1 (−33.2–7.1)^(1)^
Dyslipidemia (%)	20 (35.7)	6 (42.8)	7.1 (−18.0–34.2)^(1)^
Diabetes (%)	5 (8.9)	2 (14.3)	5.4 (−9.2–31.5)^(1)^
Tobacco (%)	28 (50.0)	6 (42.8)	−7.1 (−32.1–20.5)^(1)^
CAD (%)	3 (5.4)	0 (0.0)	−5.4 (−14.6–16.4)^(1)^
CVD (%)	3 (5.4)	0 (0.0)	−5.4 (−14.6–16.4)^(1)^
Blood parameters			
Hemoglobin, g/dl	11.8±1.4	11.44±2.1	−0.4 (−1.4–0.4)
C-reactive protein, mg/L	0.0 (0.0–6.0)	4.5 (0.0–9.7)	0.0 (0.0–5.0)^(2)^
Albumin, g/L	44.0 (42.0–46.0)	43.5 (39.0–45.0)	−1.0 (−3.0–1.0)^(2)^
Spirometry			
FEV_1_, % predicted	89.1±18.6	83.9±21.5	−5.2 (−19.7–3.6)
FEV_1_/FVC	75 (72–83)	76 (68.8–77.3)	−2.0 (−7.0–2.0)^(2)^
Arterial compliance			
PWV, m/s	7.4 (6.4–8.8)	8.2 (7.4–10.3)	0.9 (−0.2–2.0)^(2)^
Augmentation index, %	20.6±15.3	26.5±12.8	5.9 (−3.7–14.0)
CPET variables			
VO_2_AT, ml/min/kg	12.5±2.2	9.7±1.7	−2.8 (−4.1– −1.6)
VO_2_peak, ml/min/kg	22.1±5.8	16.8±4.3	−5.3 (−8.6– −2.0)
Oxygen pulse (ml O_2_/min)	11.8±4.5	9.0±2.4	−2.8 (−5.3– −0.3)
VE/VCO_2_ slope	31.2±6.6	33.9±5.2	2.7 (−1.2–6.4)

Data are mean ± SD, median (IQR) or frequencies (%). BMI, body mass index; CI, Confidence interval; CKD, chronic kidney disease; CAD, coronary artery disease; CVD, cerebrovascular disease; FEV_1_, forced expiratory volume in 1 sec; FVC, full vital capacity; PWV, carotid-femoral pulse wave velocity; CPET, cardiopulmonary exercise testing; VO_2_AT, anaerobic threshold; VO_2_peak, oxygen consumption at maximal exercise. VE/VCO_2_ (ventilatory efficiency) measured from the start of unloaded pedaling to maximal exercise. ^(1)^ Differences for non-continuous variables are expressed as relative frequency differences with corresponding Wilson Score confidence limits. ^(2)^ Differences for non-normal continuous variables are expressed as Hodges-Lehmann location shifts and Hodges-Lehmann confidence limits.

Admission to CCU within 24 hours following transplantation was documented in twelve patients whilst two patients had escalation of care to CCU more than a week following transplantation (day 10 and day 12 respectively). Hemodynamic instability (systolic arterial blood pressure ≤85 mmHg) accounted for all CCU admissions including two patients who also had sepsis (Gram negative bacteremia). 71.4% of CCU admitted patients received intravenous vasopressors whilst four patients had intra-arterial blood pressure and central venous pressure measurements to guide volume expansion without the eventual need for vasoactive drugs. There were no ischemic or arrhythmic ECG changes during the peri- and post-operative period; and at 60 days follow-up in all patients. The average duration of stay for those admitted to CCU was 3 days and this group had a longer hospital stay following transplantation (9 days, IQR: 7–15) versus those that were not admitted to CCU (7 days, IQR: 6–8; p = 0.02). No major cardiovascular events (death, myocardial infarction or non-fatal angina) were recorded at 60 days post transplantation.

### LV Morphology among Kidney Transplant Recipients

**Figure 1:**
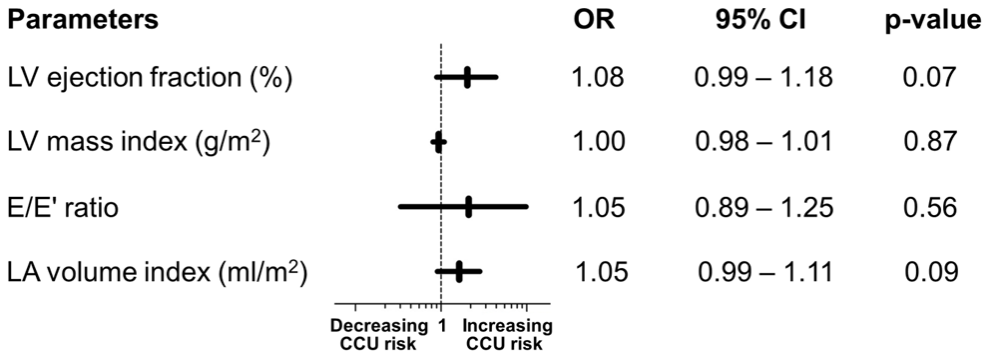
Univariate logistic regression of major echocardiographic parameters and CCU admission. LVEF, LV ejection fraction; LVMI, LV mass index corrected to body surface area; E/E’, ratio of early transmitral flow velocity to annular mitral velocity (averaged of septal and lateral); LAVI, left atrial volume index corrected to body surface area. OR, odds ratio; Horizontal bars represent the 95% CI. One metric unit increase in the parameters is associated with the odds of CCU admission.

Kidney transplant recipients had a mean LVEF of 59.9±9% and the echocardiographic indices were not statistically different between patients who were in CCU and those that did not require admission to CCU (*Table 2* *)*. Univariate logistic regression modeling did not yield a significant association between preoperative LVEF, LV mass, LV filling pressure (diastolic compliance) or LA volume with the risk of CCU admission (*Figure 1*).

**Table 2:** Echocardiographic indices for all patients.

Indices	All patients (N = 70)	Non-CCU patients (N = 56)	CCU patients (N = 14)	p –value
LVEF, %	59.9±9	58.8±8.9	63.4±8.2	0.08
Fractional shortening, %	31.7±6.6	31.7±6.7	31.8±6.3	0.92
LA diameter, cm	3.5±0.7	3.5±0.7	3.5±0.6	0.89
LAVI, mL/m^2^	25.4±10.2	24.4±7.7	29.8±17.2	0.07
LVMI, g/m^2^	118.8±42.6	119.2±39.5	117±56.4	0.87
LVH, n (%)	30 (42.9)	25 (44.6)	5 (35.7)	0.55
LV mass categorization				0.80
Normal, n (%)	13 (18.6)	11 (20)	2 (13.3)	
Concentric remodelling, n (%)	27 (38.6)	20 (36.4)	7 (46.7)	
Concentric LVH, n (%)	23 (32.9)	19 (33.9)	4 (28.6)	
Eccentric LVH, n (%)	7 (10)	6 (10.9)	1 (6.7)	
E/A	1.1±0.4	1.1±0.4	1±0.4	0.48
Deceleration time, ms	216.6±49	215.3±51	221.5±43	0.62
IVRT, ms	91±26.8	91.1±24.5	90.5±36.7	0.94
E/E’	8.3±3.2	8.2±3.4	8.8±2.7	0.57

Data are mean ± SD or frequencies (%). Analysed using independent samples t-test or χ^2^.LVEF, LV ejection fraction; LA, left atrium; LAVI, left atrial volume index corrected to body surface area; LVMI, LV mass index corrected to body surface area; LVH, left ventricular hypertrophy; E/A, the ratio of peak early to late transmitral ventricular filling velocities; IVRT, isovolumic relaxation time; E/E’, ratio of early transmitral flow velocity to annular mitral velocity (averaged of septal and lateral).

### Functional Measures of Cardiovascular Reserve

**Figure 2:**
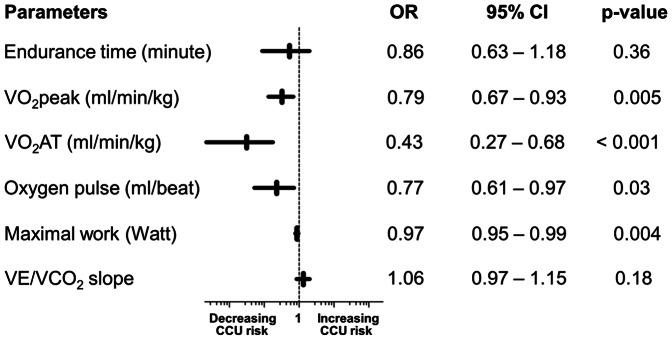
Univariate logistic regression of CPET derived measures of cardiovascular reserve and CCU admission. VO_2_peak, oxygen consumption at maximal exercise; VO_2_AT, anaerobic threshold; VE/VCO_2_ (ventilatory efficiency) measured from the start of unloaded pedaling to maximal exercise. OR, odds ratio; Horizontal bars represent the 95% CI. One metric unit increase in the parameters is associated with the odds of CCU admission. *p<0.05.

**Figure 3:**
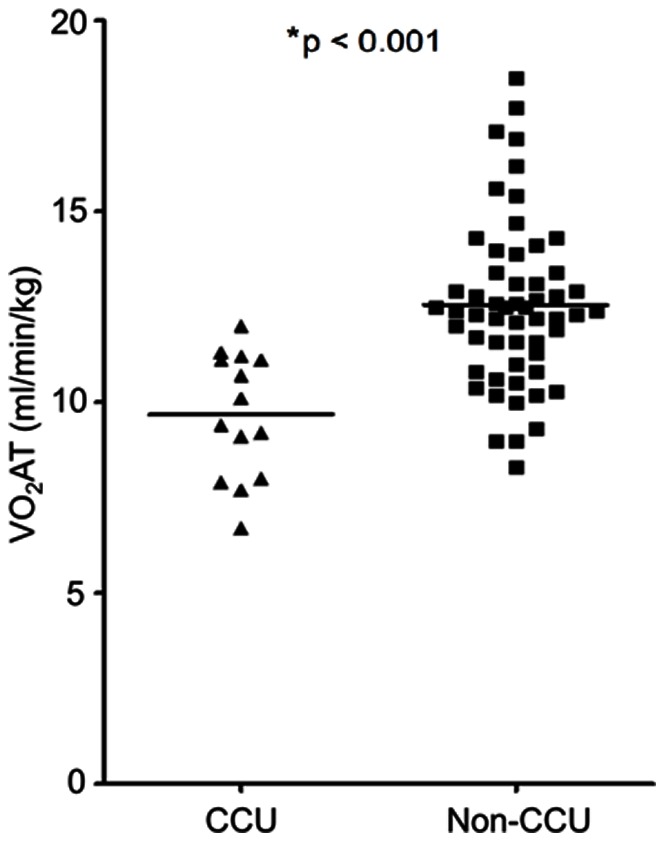
Anaerobic threshold measurements in CCU admitted and non CCU patients. Horizontal lines refers to mean values of anaerobic threshold (VO_2_AT). Squares are VO_2_AT of non CCU patients and triangles are VO_2_AT of CCU admitted patients. *The p-value of the difference in the mean values of VO_2_AT between CCU admitted and non CCU patients.

All seventy patients completed maximal exercise testing, accompanied by a mean RER of 1.25±0.12. The mean RER at the point of VO_2_AT was 0.88±0.07. The mean duration of freewheel and loaded pedaling was 10.50±2.02 minutes. Univariate logistic regressions for CPET variables’ association with CCU admission are summarized in *Figure 2*. Low levels of VO_2_AT, VO_2_max, oxygen pulse and maximum work rate were significantly associated with admission to CCU following transplantation. Measure of VO_2_AT was strongly correlated with VO_2_peak (r = 0.77, 95% CI 0.65–0.85), oxygen pulse (r = 0.54, 95% CI 0.35–0.69) and maximum work rate (r = 0.55, 95% CI 0.36–0.69) (*Table 3*). The mean VO_2_AT was significantly lower in the CCU admitted compared to non CCU patients (p<0.001) (*Table 1* and *Figure 3*).

**Table 3:** Correlation between VO_2_AT and other CPET-derived indices of cardiovascular reserve (n = 70).

Parameters	R	95% CI	p – value
VO_2_peak (ml/min/kg)	0.77	0.65–0.85	<0.001*
Oxygen pulse (ml of O_2_/beat)^a^	0.54	0.35–0.69	0.02*
Maximal work (Watt)	0.55	0.36–0.69	0.01*

a Logarithmic transformation applied for analysis; r, Pearson’s correlation coefficient; Fisher’s z-transformation has been used to derive CIs and p-values under the null hypothesis H_0:_ r = 0.3.

* p<0.05.

### Predictors of CCU Admission

**Figure 4:**
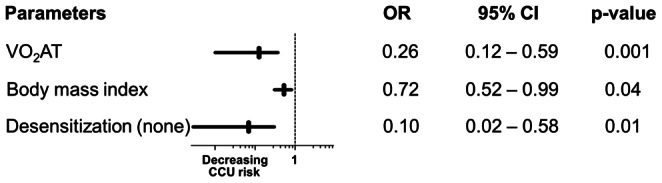
Final risk-prediction model based on 3 predictors of CCU admission. One metric unit increase in the parameters (except desensitization status) is associated with the odds of CCU admission. *Desensitization (none) = No desensitization therapy is associated with decrease odds in CCU admission. OR, odds ratio; Horizontal bars represent the 95% CI.

**Figure 5:**
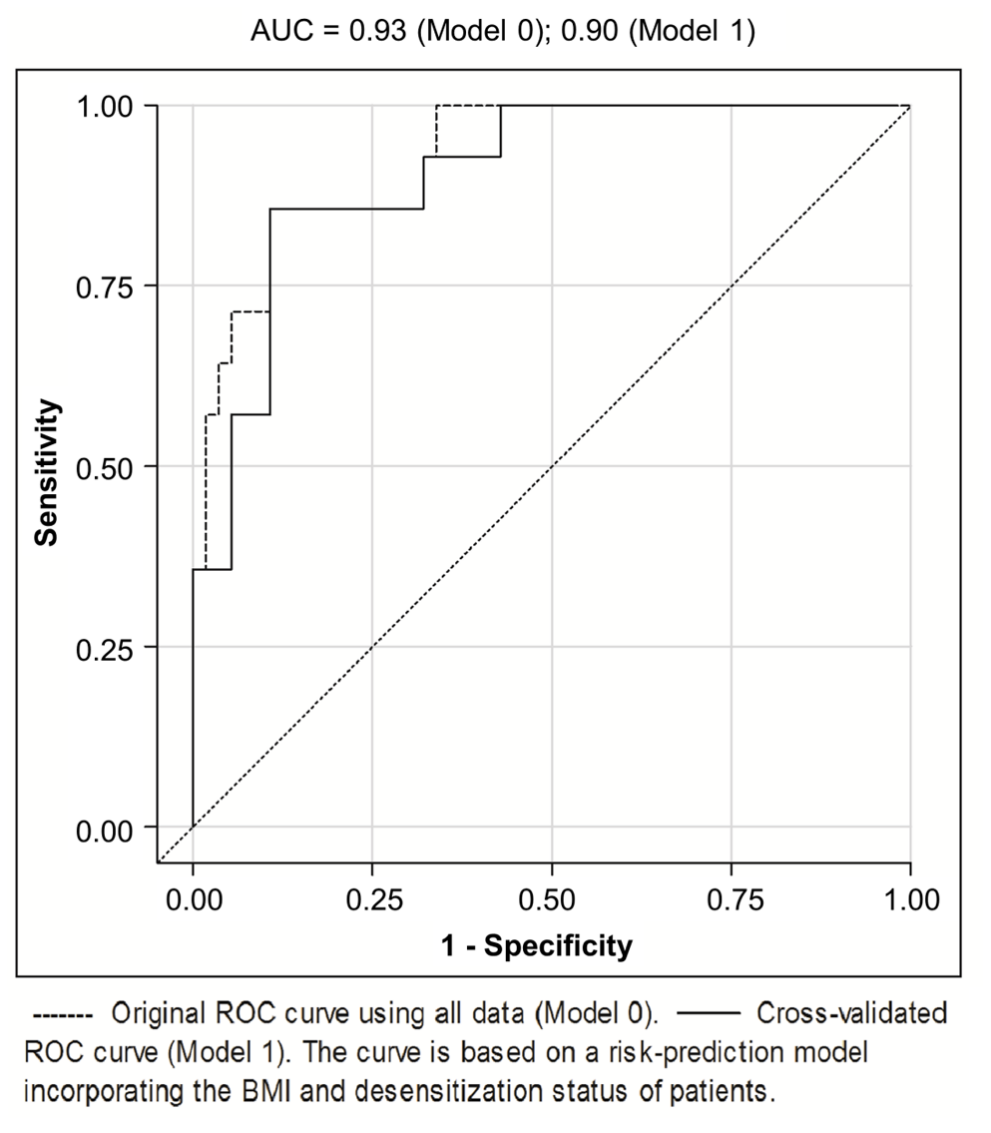
Accuracy in Predicting CCU Admission of the Final Model using Receiver Operator Characteristics (ROC) Curves.

To derive a prognostic model for CCU admission, multiple logistic regression models were fitted. Predictors which were significant in the univariate analysis were admitted to the multivariate modeling. Dialysis vintage and BMI are known confounders of cardiovascular and postoperative morbidity [25]–[27], and were therefore included in the initial set of risk predictors. Known cardiovascular risk factors were included in the variable selection process so that a total of 11 variables were considered. The established risk factors CVD and CAD (CVD/CAD) were omitted as odds ratio estimates are unobtainable due to the absence of prior history of CVD/CAD in CCU admitted patients. *Table 4* shows the predictor’s ORs and 95% CIs estimated from univariate and multivariate models. Our primary research hypothesis concerned the association of VO_2_AT and CCU admission. Therefore, and because of its high reproducibility [28] and strong correlation (multicolinearity) with the VO_2_max, oxygen pulse and maximal work rate (*Table 3*), VO_2_AT was selected for the multivariate modeling and other significant CPET variables were not. Though insignificant in the univariate analysis, patients with a higher BMI had reduced odds of CCU admission in the multivariate analysis (adjusted OR = 0.72, 95% CI 0.52–0.99, p = 0.04). *Figure 4* shows preoperative variables included in a multivariate model by variable selection procedures. The final risk prediction model consists of 3 predictors: VO_2_AT, BMI and desensitization status; model-building strategies forward selection, backwards elimination and stepwise variable selection resulted in the same model. The Hosmer-Lemeshow test is not significant, indicating that there is no evidence for lack of fit in the final multiple logistic regression model (chi-square = 6.71, degree of freedom = 8, p = 0.57). The area under the ROC curve is 0.93, indicating a very high probability of correctly classifying a randomly chosen pair consisting of a patient who requires CCU admission and a patient who does not (*Figure 5*). Using cross-validation to obtain an unbiased estimate results in an AUC of 0.90. An equation based on the model that allows estimation of the probability of CCU admission following kidney transplantation is described as follows:-.

**Table 4:** Associations between preoperative anaerobic threshold, clinical variables, arterial compliance and CCU admission (n = 70).

Parameters	Univariate† OR (95% CI)	p-value	First Model‡ OR (95% CI)	p-value	Final Model‡ OR (95% CI)	p-value
VO_2_AT, ml/min/kg	0.43 (0.27–0.68)	<0.001*	0.33 (0.14–0.78)	0.01*	0.26 (0.12–0.59)	0.001*
BMI, kg/m^2^	0.98 (0.83–1.16 )	0.82	0.70 (0.45–1.08)	0.11	0.72 (0.52–0.99)	0.04*
Desensitization (none)	0.11 (0.03–0.40)	0.001*	0.05 (0.01–0.49)	0.01*	0.10 (0.02–0.58)	0.01*
Age, year	1.05 (1.01–1.10)	0.02*	1.07 (0.97–1.17)	0.17	–	–
Gender (male)	0.19 (0.05–0.69)	0.67	0.47 (0.04–5.32)	0.54	–	–
Dialysis vintage, months	1.00 (0.99–1.01)	0.44	0.99 (0.98–1.01)	0.65	–	–
Hypertension	0.46 (0.08–2.82)	0.40	0.30 (0.02–4.67)	0.39	–	–
Dyslipidemia	1.35 (0.41–4.44)	0.62	1.11 (0.14–8.66)	0.92	–	–
Diabetes	1.77 (0.29–1.84)	0.55	0.29 (0.02–5.16)	0.39	–	–
PWV, m/s	1.08 (0.89–1.31)	0.45	–	–	–	–
Augmentation index, %	1.03 (0.98–1.07)	0.25	–	–	–	–

† Univariate logistic regression.

‡ Multiple (binary) logistic regression modeling.

* p<0.05.

#### Calculation of the predicted risk using patient’s data and multivariate logistic regression model

The odds of admission to CCU following kidney transplantation is calculated as *odds* = exp[27.003+ (−1.334×VO_2_AT)+(−0.334×BMI)+(−2.342× Desensitization status)]. The measured values are entered for the risk variables VO_2_AT and BMI, whilst desensitization status is coded as either 1 (yes) or 0 (no). The predicted risk (probability) of CCU admission following kidney transplantation is calculated as *odds*/1+ *odds*.

Considering the variable VO_2_AT univariately as a predictor yields an optimal cut-off of VO_2_AT = 11.31 ml/min/kg for distinguishing between the CCU admitted and non-admitted populations (Youden index J = 0.68). This translates into a sensitivity of 92.9% and a specificity of 75.0% at this cut-off point.

## Discussion

This is the first prospective study to demonstrate an independent association between objective indices of cardiovascular reserve, in particular the VO_2_AT, measured preoperatively and CCU admission following kidney transplantation. In the preoperative assessment setting of major intra-abdominal surgery (all previous studies precluded kidney transplantation), three physiological CPET variables have been shown effectively to identify high risk patients: VO_2_AT [15], [16], VO_2_peak [18] and ventilatory equivalent for carbon dioxide (VE/VCO_2_) [16], [17]. This study identifies similar importance of VO_2_peak and VO_2_AT, with the additional measures of oxygen pulse and maximal work rate as markers of high perioperative risks (*Figure 2*). The index of VE/VCO_2_ appears not to have any significant preoperative risk predicting strength among CKD patients. VO_2_AT is defined as the point during incremental exercise when oxygen demand exceeds oxygen delivery, thus causing the anaerobic metabolism to make up the deficit, with a consequential rise in serum lactate concentration. It is a measure of the ability of the cardiovascular system to deliver adequate oxygen to tissue and possesses the advantage over VO_2_peak, of being independent of patient motivation or premature termination of test [28]. Among the CPET variables in this study, measure of sustainable oxygen consumption (VO_2_AT) carries the strongest risk predicting strength independently (*Figure 2* *)* and remains significant after adjusting for age and gender (*Table 4*), attesting to the study’s hypothesis that reduced objective measure of cardiovascular reserve is associated with an increased perioperative morbidity.

In contrast with other studies [15]–[18], low VO_2_AT among kidney transplant recipients does not carry any risk of death or cardiac ischemia at 60 days following surgery. This is despite the fact that a similar proportion of patients (33%) who underwent surgery had an VO_2_AT of less than 11 ml/min/kg in this study compared with previous studies, such as 29% in the one by Older and colleagues [15]. This suggests that the prognostic values of VO_2_AT in other major intra-abdominal studies may not be transferable to kidney transplantation. The study also shows no associations between the static measures of cardiac function (echocardiography) or aortic compliance and perioperative risk among CKD patients. Additionally, there were no events of cardiac ischemia during the perioperative period to 60 days following surgery. The relevance of these is in keeping with the findings of a recent preoperative (kidney transplant) cardiac assessment study that demonstrated not only the low incidence of cardiac ischemia perioperatively among high cardiac risk cohort but also the lack of prognostic abilities of tools such as myocardial scintigraphy, dobutamine stress echocardiography or coronary angiogram (with subsequent revascularization if positive stress test) in reducing perioperative cardiac events [29].

In antibody incompatible transplantations, patients receive more cumulative immunosuppression treatment, including plasmapharesis and anti-thymocyte globulin [19], [30], which increase the risk of sepsis. However, among the eight desensitized patients admitted to CCU, only one required admission following a development of neutropenic sepsis and hypotension, 10 days following kidney transplantation. Adverse cardiovascular effects of the standard oral immunosuppressive therapies usually occur over time and are unlikely to have any direct impact on perioperative outcome [31]. However, the insult of sepsis can expose the limits of patient’s cardiovascular reserve. The hemodynamic profile of sepsis includes loss of vascular responsiveness and decrease venous return to the heart resulting in a decrease in cardiac output [32], [33]. Therefore, rapid fluid therapy at the early stage of sepsis is vital. It also modulates inflammation and prevents further myocardial depression caused by inflammatory cytokines [34]. Hypotension after adequate restoration of LV preload with volume expansion could largely be related to impairment of cardiovascular contractility [35], [36]. It could also be speculated that, the subclinical effects of myocardial stunning on the LV, induced by hemodialysis [37], [38] could be exaggerated by additional high volume plasmapheresis therapy [19], [30] causing further reduction to the functional cardiovascular reserve, that were measured prior to the start of desensitization treatment. Hence, these crucial pre-operative treatment strategies could have altered and increased patients’ surgical cardiac risk status at the time of surgery. Furthermore, these patients also carried the burden of disease chronicity greater than those who did not require desensitization [dialysis vintage: 26 (15–106) vs. 6 (0–32) months, p = 0.01; CKD duration: 192 (84–252) vs. 120 (42–211) months, p = 0.08). The effect of having no dialysis prior to kidney transplantation on perioperative outcome was examined by ordering patients into 2 groups: pre-dialysis and dialysis dependent. Even though dialysis vintage did not appear to contribute to the risk of perioperative need for CCU, patients who were transplanted pre-emptively had a lower risk of CCU admission than those who were dialysis dependent (*Table 1*).

PWV was found to be insignificant in predicting perioperative morbidity in this study (*Table 4* *)*. This could be due to the fact that PWV provides information on the stiffness of the local vessels being studied rather than the systemic arterial stiffness [39]. The latter along with cardiac output ( =  stroke volume × heart rate) are determinants of the mean arterial pressure which is an indicator of hemodynamic stability. Although this finding also reflects the limitation of using single surrogate marker as a risk predictor, the value of PWV should not be underestimated due to the recognized pathophysiological role of central arterial stiffness on LV afterload [40]. The outgoing systolic pressure wave that traverses the stiffened aorta is reflected early causing an increase in the augmentation and central pulse pressures. The premature reflection of the systolic waveform also shortens the critical diastolic phase during which the coronaries are being perfused with the end result of myocardial depression [40], [41]. This may explain the inverse correlation between PWV and AT as a global measure of functional cardiovascular reserve in our cohort (Pearson correlation, r =  −0.32, p = 0.007).

Many nephrologists and transplant surgeons have concerns about the higher rates of postoperative complications in overweight patients with their possible effects on survival outcomes and hence patients with BMI greater than 35 kg/m^2^ are suspended from transplantation. However, it is important to note that this study has shown that lower BMI is associated with significant perioperative morbidity after adjusting for important covariates as shown in *Table 4*. This confirms the findings of other observational studies that low BMIs, often associated with low VO_2_peak in hemodialysis patients [42], also carries a high morbidity risk among kidney transplant recipients [43]. Here, it may also be important to recognize the relevance of the reverse epidemiology observed in end-stage renal disease patients in whom high BMI is paradoxically protective [44], [45].

The prognostic value of VO_2_AT is demonstrated in the risk-prediction model that included both the BMI and desensitization status (*Figure 5*). The AUC was 0.93 for the ROC curve using all the available data to estimate these parameters (Model 0) and using cross validation (Model 1), the AUC was still very high albeit reduced to 0.90. These findings demonstrate that in combination, these 3 variables perform well as a preoperative risk stratification tool for predicting perioperative morbidity among kidney transplant recipients leading to CCU admission.

Even though the design of this study was prospective, the limitation was the relatively small sample size. The study was also not powered for mortality. However, there were no deaths despite the lowest measured VO_2_AT being 6.7 ml/min/kg. The majority of CKD patients in this study were kidney recipients of living donor and the results presented here may not be reflective in a study cohort that consisted only of deceased kidney recipients. However, the risk may be comparable to patient population undergoing antibody removal. On the intention-to-treat basis, this study was designed to include all patients including those with high immunological risk. Therefore, the relatively higher than expected rate of CCU admission in our cohort may be due to the large numbers of antibody-incompatible kidney transplants performed in our center compared to an average kidney transplant unit. Finally, questions remain, whether the CPET derived markers of cardiovascular reserve could be modified or improved (example, with individualized exercise training) with a subsequent reduction in the perioperative risk associated with kidney transplantation or if the measure of VO_2_AT could improve the transplant work-up process and increase the inclusion of patients into the transplant programme, who are otherwise excluded based on current clinical criterions.

### Conclusions

This is the first prospective study to demonstrate the usefulness of CPET as a preoperative risk stratification tool for patients undergoing kidney transplantation. The study suggests that objective measures of functional cardiovascular reserve, in particular the VO_2_AT, have the potential to predict perioperative morbidity in kidney transplant recipients. A further study of larger sample is required to validate the findings.

## References

[1] Meier-Kriesche HU, Schold JD, Srinivas TR, Reed A, Kaplan B (2004) Kidney transplantation halts cardiovascular disease progression in patients with end-stage renal disease. Am J Transplant 4: 1662–1668. 15367222 10.1111/j.1600-6143.2004.00573.x

[2] Sadaghdar H, Chelluri L, Bowles SA, Shapiro R (1995) Outcome of renal transplant recipients in the ICU. Chest 107: 1402–1405. 7750338 10.1378/chest.107.5.1402

[3] Older P, Smith R (1988) Experience with the preoperative invasive measurement of haemodynamic, respiratory and renal function in 100 elderly patients scheduled for major abdominal surgery. Anaesth Intensive Care 16: 389–395. 3232797 10.1177/0310057X8801600402

[4] Raggi P, Boulay A, Chasan-Taber S, Amin N, Dillon M, et al (2002) Cardiac calcification in adult hemodialysis patients. A link between end-stage renal disease and cardiovascular disease? J Am Coll Cardiol 39: 695–701. 11849871 10.1016/s0735-1097(01)01781-8

[5] Aoki J, Ikari Y, Nakajima H, Mori M, Sugimoto T, et al (2005) Clinical and pathologic characteristics of dilated cardiomyopathy in hemodialysis patients. Kidney Int 67: 333–340. 15610259 10.1111/j.1523-1755.2005.00086.x

[6] Himmelfarb J, Ikizler TA (2010) Hemodialysis. N Engl J Med 363: 1833–1845. 21047227 10.1056/NEJMra0902710

[7] Farr MJ, Lang CC, Lamanca JJ, Zile MR, Francis G, et al (2008) Cardiopulmonary exercise variables in diastolic versus systolic heart failure. Am J Cardiol 102: 203–206. 18602522 10.1016/j.amjcard.2008.03.041

[8] Kitzman DW, Higginbotham MB, Cobb FR, Sheikh KH, Sullivan MJ (1991) Exercise intolerance in patients with heart failure and preserved left ventricular systolic function: failure of the Frank-Starling mechanism. J Am Coll Cardiol 17: 1065–1072. 2007704 10.1016/0735-1097(91)90832-t

[9] Witte KK, Nikitin NP, Cleland JG, Clark AL (2006) Excessive breathlessness in patients with diastolic heart failure. Heart 92: 1425–1429. 16621875 10.1136/hrt.2005.081521 PMC1861064

[10] Wasserman K (1997) Diagnosing cardiovascular and lung pathophysiology from exercise gas exchange. Chest 112: 1091–1101. 9377922 10.1378/chest.112.4.1091

[11] Cooper CB, Storer TW (2010) Exercise testing and interpretation: Cambridge University Press, New York.

[12] Guazzi M, Adams V, Conraads V, Halle M, Mezzani A, et al (2012) EACPR/AHA Scientific Statement. Clinical recommendations for cardiopulmonary exercise testing data assessment in specific patient populations. Circulation 126: 2261–2274. 22952317 10.1161/CIR.0b013e31826fb946 PMC4777325

[13] Belardinelli R, Lacalaprice F, Carle F, Minnucci A, Cianci G, et al (2003) Exercise-induced myocardial ischaemia detected by cardiopulmonary exercise testing. Eur Heart J 24: 1304–1313. 12871687 10.1016/s0195-668x(03)00210-0

[14] Pinkstaff S, Peberdy MA, Fabiato A, Finucane S, Arena R (2010) The clinical utility of cardiopulmonary exercise testing in suspected or confirmed myocardial ischemia. Am J Lifestyle Med 4: 327–348.

[15] Older P, Hall A, Hader R (1999) Cardiopulmonary exercise testing as a screening test for perioperative management of major surgery in the elderly. Chest 116: 355–362. 10453862 10.1378/chest.116.2.355

[16] Wilson RJ, Davies S, Yates D, Redman J, Stone M (2010) Impaired functional capacity is associated with all-cause mortality after major elective intra-abdominal surgery. Br J Anaesth 105: 297–303. 20573634 10.1093/bja/aeq128

[17] Carlisle J, Swart M (2007) Mid-term survival after abdominal aortic aneurysm surgery predicted by cardiopulmonary exercise testing. Br J Surg 94: 966–969. 17440956 10.1002/bjs.5734

[18] McCullough PA, Gallagher MJ, Dejong AT, Sandberg KR, Trivax JE, et al (2006) Cardiorespiratory fitness and short-term complications after bariatric surgery. Chest 130: 517–525. 16899853 10.1378/chest.130.2.517

[19] Higgins R, Lowe D, Hathaway M, Williams C, Lam FT, et al (2011) Human leukocyte antigen antibody-incompatible renal transplantation: excellent medium-term outcomes with negative cytotoxic crossmatch. Transplantation 92: 900–906. 21968524 10.1097/TP.0b013e31822dc38d

[20] Gardner-Thorpe J, Love N, Wrightson J, Walsh S, Keeling N (2006) The value of Modified Early Warning Score (MEWS) in surgical in-patients: a prospective observational study. Ann R Coll Surg Engl 88: 571–575. 17059720 10.1308/003588406X130615 PMC1963767

[21] (2007) National Institute for Health and Clinical Excellence. Acutely ill patients in hospital: recognition of and response to acute illness in adults in hospital.. 21204323

[22] Wasserman K, Hansen JE, Sue DY, Stringer WW, Whipp BJ (2005) Principles of exercise testing and interpretation : including pathophysiology and clinical applications. 4 ed. Philadelphia: Lippincott Williams & Wilkins.

[23] Cheitlin MD, Armstrong WF, Aurigemma GP, Beller GA, Bierman FZ, et al (2003) ACC/AHA/ASE 2003 Guideline Update for the Clinical Application of Echocardiography: summary article. A report of the American College of Cardiology/American Heart Association Task Force on Practice Guidelines (ACC/AHA/ASE Committee to Update the 1997 Guidelines for the Clinical Application of Echocardiography). J Am Soc Echocardiogr 16: 1091–1110. 14566308 10.1016/S0894-7317(03)00685-0

[24] Perkins NJ, Schisterman EF (2006) The inconsistency of “optimal” cutpoints obtained using two criteria based on the receiver operating characteristic curve. Am J Epidemiol 163: 670–675. 16410346 10.1093/aje/kwj063 PMC1444894

[25] Chang SH, Coates PT, McDonald SP (2007) Effects of body mass index at transplant on outcomes of kidney transplantation. Transplantation 84: 981–987. 17989603 10.1097/01.tp.0000285290.77406.7b

[26] Habedank D, Kung T, Karhausen T, von Haehling S, Doehner W, et al (2009) Exercise capacity and body composition in living-donor renal transplant recipients over time. Nephrol Dial Transplant 24: 3854–3860. 19736242 10.1093/ndt/gfp433

[27] Meier-Kriesche HU, Port FK, Ojo AO, Rudich SM, Hanson JA, et al (2000) Effect of waiting time on renal transplant outcome. Kidney Int 58: 1311–1317. 10972695 10.1046/j.1523-1755.2000.00287.x

[28] Gitt AK, Wasserman K, Kilkowski C, Kleemann T, Kilkowski A, et al (2002) Exercise anaerobic threshold and ventilatory efficiency identify heart failure patients for high risk of early death. Circulation 106: 3079–3084. 12473555 10.1161/01.cir.0000041428.99427.06

[29] Aalten J, Peeters SA, van der Vlugt MJ, Hoitsma AJ (2011) Is standardized cardiac assessment of asymptomatic high-risk renal transplant candidates beneficial? Nephrol Dial Transplant 26: 3006–3012. 21321004 10.1093/ndt/gfq822

[30] Higgins R, Lowe D, Hathaway M, Lam FT, Kashi H, et al (2010) Double filtration plasmapheresis in antibody-incompatible kidney transplantation. Ther Apher Dial 14: 392–399. 20649760 10.1111/j.1744-9987.2010.00821.x

[31] Miller LW (2002) Cardiovascular toxicities of immunosuppressive agents. Am J Transplant 2: 807–818. 12392286 10.1034/j.1600-6143.2002.20902.x

[32] Snell RJ, Parrillo JE (1991) Cardiovascular dysfunction in septic shock. Chest 99: 1000–1009. 2009750 10.1378/chest.99.4.1000

[33] Parrillo JE (1993) Pathogenetic mechanisms of septic shock. N Engl J Med 328: 1471–1477. 8479467 10.1056/NEJM199305203282008

[34] Kumar A, Haery C, Parrillo JE (2000) Myocardial dysfunction in septic shock. Crit Care Clin 16: 251–287. 10768082 10.1016/s0749-0704(05)70110-x

[35] Rivers EP, Coba V, Whitmill M (2008) Early goal-directed therapy in severe sepsis and septic shock: a contemporary review of the literature. Curr Opin Anaesthesiol 21: 128–140. 18443478 10.1097/ACO.0b013e3282f4db7a

[36] Rivers EP, Ahrens T (2008) Improving outcomes for severe sepsis and septic shock: tools for early identification of at-risk patients and treatment protocol implementation. Crit Care Clin 24: S1–47. 18634996 10.1016/j.ccc.2008.04.002

[37] Burton JO, Jefferies HJ, Selby NM, McIntyre CW (2009) Hemodialysis-induced repetitive myocardial injury results in global and segmental reduction in systolic cardiac function. Clin J Am Soc Nephrol 4: 1925–1931. 19808220 10.2215/CJN.04470709 PMC2798881

[38] Burton JO, Jefferies HJ, Selby NM, McIntyre CW (2009) Hemodialysis-induced cardiac injury: determinants and associated outcomes. Clin J Am Soc Nephrol 4: 914–920. 19357245 10.2215/CJN.03900808 PMC2676185

[39] Mackenzie IS, Wilkinson IB, Cockcroft JR (2002) Assessment of arterial stiffness in clinical practice. QJM 95: 67–74. 11861952 10.1093/qjmed/95.2.67

[40] London GM (2003) Cardiovascular calcifications in uremic patients: clinical impact on cardiovascular function. J Am Soc Nephrol 14: S305–309. 12939386 10.1097/01.asn.0000081664.65772.eb

[41] O’Rourke MF, Kelly RP (1993) Wave reflection in the systemic circulation and its implications in ventricular function. J Hypertens 11: 327–337. 8390498 10.1097/00004872-199304000-00001

[42] Sezer S, Elsurer R, Ulubay G, Ozdemir FN, Haberal M (2007) Factors associated with peak oxygen uptake in hemodialysis patients awaiting renal transplantation. Transplant Proc 39: 879–882. 17524838 10.1016/j.transproceed.2007.02.013

[43] Meier-Kriesche HU, Arndorfer JA, Kaplan B (2002) The impact of body mass index on renal transplant outcomes: a significant independent risk factor for graft failure and patient death. Transplantation 73: 70–74. 11792981 10.1097/00007890-200201150-00013

[44] Kalantar-Zadeh K, Block G, Humphreys MH, Kopple JD (2003) Reverse epidemiology of cardiovascular risk factors in maintenance dialysis patients. Kidney Int 63: 793–808. 12631061 10.1046/j.1523-1755.2003.00803.x

[45] Kalantar-Zadeh K, Kopple JD, Kilpatrick RD, McAllister CJ, Shinaberger CS, et al (2005) Association of morbid obesity and weight change over time with cardiovascular survival in hemodialysis population. Am J Kidney Dis 46: 489–500. 16129211 10.1053/j.ajkd.2005.05.020

